# Operation for the Removal of an Ovarian Tumor, Weighing 17 Lbs.

**Published:** 1861-03

**Authors:** E. S. Cooper

**Affiliations:** Professor of Anatomy and Surgery in the Medical Department of the University of the Pacific


*Operation for the Removal of an Ovarian Tumor, weighing 17 Pounds.—Death of Patient, by subsequent Internal Hemorrhage.* By E. S. Cooper, A.M. M.D., Professor of Anatomy and Surgery in the Medical Department of the University of the Pacific.

In this case, the tumor (fibrous) was adherent, by an immense pedicle, reaching from the seat of one ovary to that of the other, and containing nearly a dozen large arteries, capable of bleeding the patient to death, almost at once, had the tumor been separated directly by the knife. It had no surrounding attachments.

It will be seen, after the detailing of this case, that the greatest care was taken to prevent hemorrhage, but, notwithstanding this, death occurred, from this cause, six hours after the operation.

Humiliating as the confession is, I must say, that two patients in three, whom I have lost after this operation, died of internal hemorrhage, after the wounds were dressed; and I wish, in this paper, to direct the attention of the medical profession particularly to the subject.

Sometimes it is a very easy matter to arrest the hemorrhage completely, but, again, there are cases in which the greatest skill may be required. I this case, there was an artery almost as large as the arteria innominata, and many others as large as the primitive carotids, entering the tumor, and this, associated with the close connection between it and the abdominal aorta, would have rendered it impossible to isolate and tie the arteries, if left until after the division of the pedicle and removal of the tumor. The pedicle was therefore, transfixed in a great many places, and tied, section by section, until at least a dozen ligatures had been pressed through it, at different points, before an attempt was made to divide the pedicle. Even then, when this was begun, though the vessels on the side where the division commenced were strangulated, so that, after a small part of the pedicle was cut across, no bleeding occurred from the proximal side. Still an active hemorrhage occurred from the distal side of the cut, showing that there were yet remaining large supply arterial trunks, not strangulated, entering the tumor. Five or six very strong ligatures were then thrown entirely around the pedicle and drawn as tightly as the strength of one man could well make them, and were held to their places, by being fastened to the ligatures that had been made to transfix it. This so far arrested the circulation that no hemorrhage occurred from the distal side of the cut, as I divided the parts cautiously and little by little. But, after all the precaution that was taken to strangulate the vessels, it was found, after the separation of the tumor, that some of them had retracted and caused a slight degree of hemorrhage. These were promptly secured, and the hemorrhage, for the time being, arrested. The wound was left open, and the bleeding surface exposed, as long as I thought it prudent to permit it, in order to show every vessel disposed to retract and bleed. But the shortness of the pedicle and its immense thickness was such, that it was impossible to have the contents of the abdominal cavity constantly retained in their proper places, and, at the same time, allow the necessary manipulations with the needle, described, without wounding them. It is seldom that the exposure of the intestines cause death, after the removal of ovarian tumors, which makes this a matter of secondary importance. And as I wish here to give my opinion upon the best means of securing success in these operations, I must say, that those which prevent hemorrhage are the first of all to be considered.

In this case, though all possible care was observed, in this respect, the patient sank, from the effects of internal hemorrhage.

I have never lost a patient from peritoneal inflammation, after this operation. All my fatal cases have occurred within twelve hours after its performance. It will, likewise, be found, by referring to the history of fatal cases, that death, generally, occurs within a few hours, and I doubt not, internal hemorrage is more often the cause than is suspected. This subject has attracted but little attention among surgeons, but it is the all-important one in these operations. This paper may, I hope, be the means of calling the attention of the surgical world to the matter, and giving it the importance it so much deserves. In this instance, I am convinced, no care or attention would have saved the patient from hemorrhage; but in others, in which I have operated previously, death resulted from hemorrhage, which the precautions observed in this case would have prevented.

Some surgeons, who appear to think everything depends upon making small incisions through the abdominal walls, and protecting the viscera from exposure to the atmospheric influence, lose sight of this important fact, and the consequence is, that their patients often lose immense amounts of blood, so much, in fact, as not unfrequently they die upon the table. Most other injuries, however severe, may sometimes be recovered from, but the loss of blood, beyond a given point, never can; and when surgeons try, by small incisions and forcibly retaining the intestines in the abdominal cavity, by which the chances of arresting hemorrhage will be greatly diminished, they are liable to change an uncertain into a certain fatality.

In all grave operations, where there are many risks to run, we may be compelled to make a choice of the least, so, in this operation, we should take those chances which enable us to avoid the greatest risk.

I learn, very recently, that Dr. Toland, of this city, has had a case of operation for the removal of a large ovarian tumor, which terminated successfully. The details of the case I am unable to give, still, in a statistical point of view, the case should be mentioned in this connection. It makes the third operation performed in California within a year, two of which terminated favorably; more than can be said of previous results in this State. It has been, thus far, much more fatal than might be expected, in our favorable climate. Though frequently performed by surgeons of skill, it has seldom been successful.

Dr. A. F. Sawyer has given the following statistical report of abdominal sections, made in California, prior to the period in which the three mentioned were performed.

" We append the following list of cases of abdominal section with results—total of eleven cases, which probably includes all the operations of this character made in California :

Seven cases of ovarian disease, of which six terminated fataly. In three of the seven cases, the wound was closed without attempt at removal of the tumor, on account of unusual complications. In one case, the contents of the cyst were purulent. In the seventh case, (Dr. Nelson's,) the patient made a perfect recovery, although the case appeared unfavorable, from extensive adhesions. One case of Cæsarian operation, (Dr. Cooper), successful. One case of fibrous tumor of uterus, (Dr. Nelson), successful. One case of carcinoma of uterus, (the case was here reported), fatal. One case of fungus hæmatodes of kidney, fatal.

These cases have generally, been in the hands of fully competent and experienced surgeons; and the fatality, as far as ovarian disease is concerned, (six out of seven cases), compares very unfavorably with the published statistical accounts of the success of this operation."—*San Francisco Medical Press.*

